# More Suits for Malpractice

**Published:** 1847-09

**Authors:** 


ART. II.—*More suits for Malpractice.*—Dr Hill, of Medina, one of the most respectable surgeons of Western New-York, has lately been tried a second time for alledged malpractice in a case of luxation of radius and ulna backwards, and the jury have a second time disagreed. It is understood that they were equally divided as to conviction or acquittal.

This is a singular case, and more than any thing which has ever come to our knowledge, exhibits the perilous condition of those who practise surgery; and unless some remedy is devised—some protection afforded against unjust prosecutions, all respectable men must be soon driven from the ranks. As the case is to be tried again (so says the plaintiff, but we doubt it) we will simply state, that the plaintiff received a severe injury on the elbow-joint, which was seen by a physician very soon after and dressed. After the lapse of some weeks, Dr. Hill was requested to see it, and, on examination, he, with Dr. Ford, pronounced it an unreduced, backward luxation of radius and ulna. The signs were too manifest to be for a moment mistaken. Drs. Hill and Ford, after advising the plaintiff of the small probability of success, attempted to reduce it, but finally gave over and left the arm as before. Dr. Hill is now charged with having *produced* the very luxation which at present exists, and which he vainly endeavored to reduce!

Now, aside of the almost utter impossibility of making such a luxation by any means when the patient is watching the muscles, and the equal improbability of their *pushing* the bones in such a direction (backwards) as to induce a luxation, when actually *pulling* to reduce them; aside of all this, we say, we know the history and the facts as elicited in the trial, and we do not hesitate to affirm that were the case presented to a jury of 1000 surgeons, or to *any* jury competent to examine such a subject, and *honest* men, the Dr. would receive the prompt acquittal of every man. More than this, we ourselves know these surgeons, who, after a careful exammination, declared it a luxation, and who made the attempt at reduction. Of the reputation of the defendant, we have spoken; and of Dr. Ford we will also say, that, as an anatomist, he has not his peer among us, and we could as soon believe that an Otis or a Lefevre would fail to know whether the tenon of a timber rested in its mortice as that Dr. Ford would fail to recognize an ancient luxation of this character. X. Y. Z.

